# A flow visualization model of duodenogastric reflux after esophagectomy with gastric interposition

**DOI:** 10.1186/1749-8090-8-192

**Published:** 2013-09-25

**Authors:** Chul-Hyun Park, Jae-Ik Lee, Jaeyong Sung, Sunghoon Choi, Kwang-Pil Ko

**Affiliations:** 1 Department of Thoracic & Cardiovascular Surgery, Gachon University Gil Hospital, Incheon, Korea; 2 Department of Mechanical Engineering, Seoul National University of Science and Technology, Seoul, Korea; 3 Department of Preventive Medicine, Gachon University of Medicine and Science, Incheon, Korea

**Keywords:** Esophageal surgery, In vitro studies, Quality of life

## Abstract

**Background:**

Our goal was to verify surgical factors that affect duodenogastric reflux (DGR) after esophagectomy through the use of a flow visualization model that would mimic an intrathoracic gastric tube.

**Methods:**

Transparent gastric tube models for different routes (retrosternal space [RS] and posterior mediastinum [PM]) were fabricated. Various distal pressures were applied to the experimental model filled with water, and the flow was recorded with a high-speed camera. The volume and maximum height of the refluxate through the pylori of two different sizes (7.5 mm, 15 mm) in two different postures (upright, semi-Fowler) was measured by analyzing the video clips.

**Results:**

For the large pylorus setting, when the pressures of 20, 30, and 40 mmHg were applied in the upright position, the volumes of the refluxate in the RS/PM tubes were 87.7 ± 1.1/96.4 ± 1.7 mL, 150.8 ± 1.1/158.0 ± 3.2 mL, and 156.8 ± 3.3/198.0 ± 4.7 mL (p < 0.05), and the maximum heights were 101.6 ± 4.8/113.4 ± 2.9 mm, 151.4 ± 2.2/165.4 ± 1.5 mm, and 166.1 ± 1.7/193.7 ± 6.6 mm (p < 0.05). The data for the small pylorus setting or in the semi-Fowler position showed similar tendencies. For any given route, posture or pressure setting, DGR in the large pylorus model was definitively higher than that for small one.

**Conclusions:**

This fluid mechanics study demonstrates posterior mediastinal gastric interposition or pyloric drainage procedure, or both, is associated with high reflux of duodenal contents.

## Background

Duodenogastric reflux (DGR) is a common pathophysiological sequela of esophagectomy with gastric interposition; it has been documented in 60 ~ 80% of patients [1]. The reflux symptoms adversely affect the quality of life in these patients [1-5]. Furthermore, there is evidence that the duodenal contents are noxious and may, in the long term, cause mucosal changes both to the gastric conduit and the esophageal remnant [1]. A truncal vagotomy that necessarily accompanies the procedure is considered the main cause of DGR. It impairs the physiological balance between propulsive activity of the antrum and pyloroduodenal resistance to gastric in and outflows of biliopancreatic secretion [6].

To eliminate this complication, some authors have advocated the omission of a pyloric drainage procedure [7], and the use of an extra-anatomical space as a route of esophageal reconstruction rather than using the posterior mediastinum [3,8]. Controversy still exists about the omission of a pyloric drainage procedure and the use of an extra-anatomical route because the evidence from the literature is conflicting [4,5,9-12]. A reason for these conflicting results is that the clinical data from human subjects is not easily reproducible due to many other factors that affect DGR and cannot be completely controlled in clinical settings.

The aim of this study was to verify the role of the route of reconstruction and a pyloric drainage procedure on postoperative DGR by using a flow visualization model of the intrathoracic stomach, which we have previously described [13].

## Methods

For the design of an intrathoracic gastric tube model, we reviewed the postoperative chest CT images of 10 patients with esophageal cancer who underwent esophagectomy with gastric interposition (5 in the retrosternal space (RS) and 5 in the posterior mediastinum (PM)) at Gachon University Gil Hospital. This study has been approved by the Institutional Review Board of Gachon University Gil Hospital.

### Data collection from CT images

**Figure 1:**
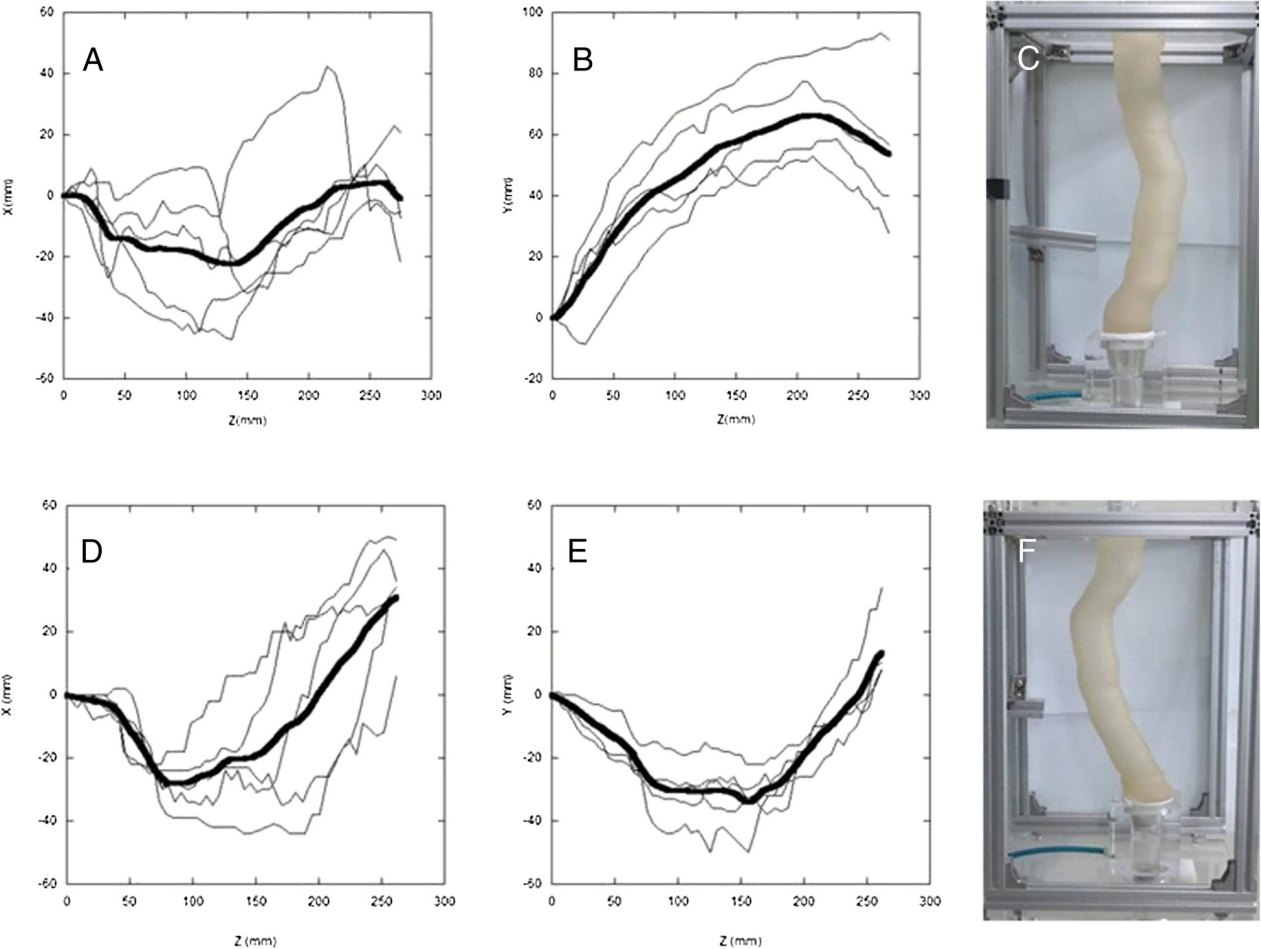
**Averaging the course of the gastric tube.** Bold lines represent the average of centroids (x, y) of 5 gastric tubes according to the vertical position (z) in each route. **A**, x in the retrosternal route. **B**, y in the retrosternal route. **C**, Retrosternal gastric tube model. **D**, x in the posterior mediastinal route. **E**, y in the posterior mediastinal route. **F**, Posterior mediastinal gastric tube model.

For each patient, all of the CT slices from the esophago-gastric anastomotic site to the level of the pylorus were taken by changing the vertical position z, and the data about the centroids (x, y) in the cross sections of the gastric tube were obtained. Then, the centroids and heights were averaged for the 5 patients in each group (Figure 1). Finally, the three-dimensional courses of the gastric tube (in RS and PM) were averaged and used for reconstructions.

### Model fabrication and setting

**Figure 2:**
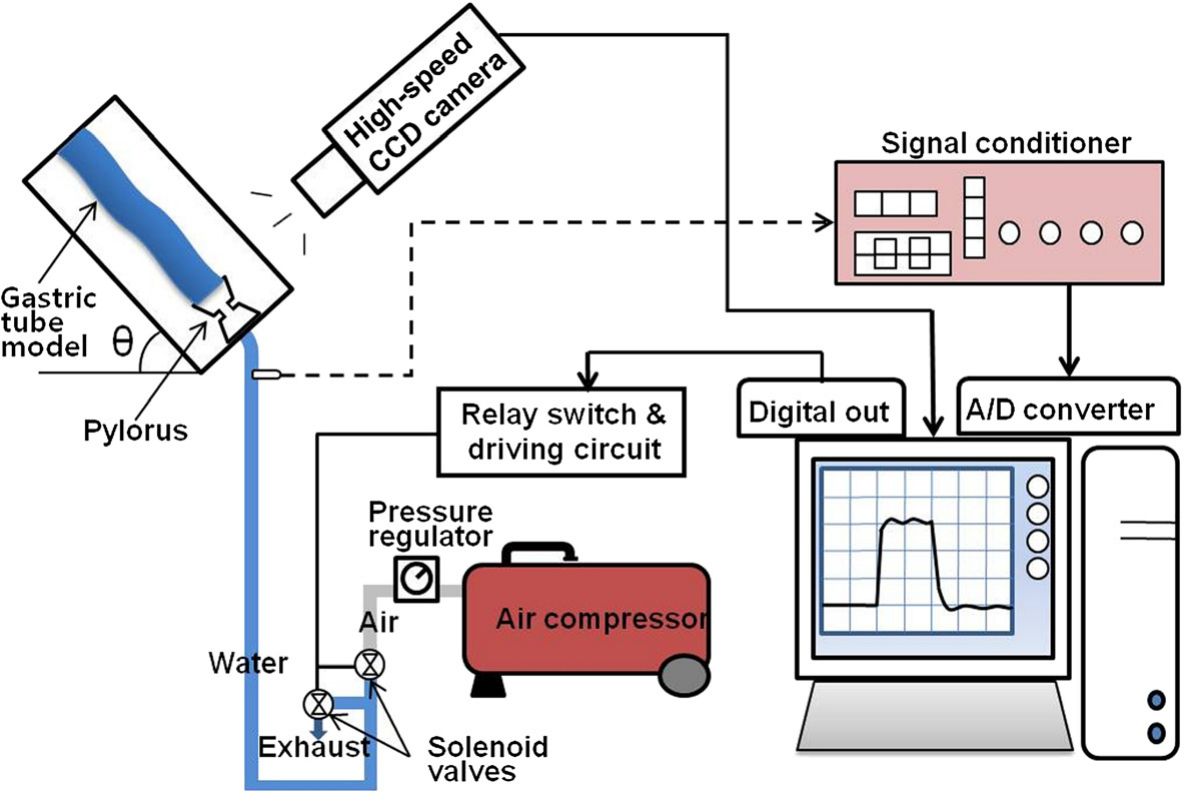
Experimental setup for measuring reflux.

The experimental models were made in the same manner as previously described by the authors [13]. For simple comparison, the passages were modeled as circular tubes with an inner diameter of 40 mm (to a thickness of 5 mm), using a rapid prototyping technique. They were connected to the pylorus models with two different diameters of pyloric opening (7.5 mm or 15 mm) (Figure 1). Figure 2 shows the experimental setup for measuring DGR. A frame supporting the gastric tube model was designed to be tilted by a hinge. An air compressor supplied distal pressure, and two solenoid valves (supply and exhaust) were used to control the width of a pressure pulse. When the supply valve was opened and the exhaust valve was closed simultaneously by a relay switch, compressed air was applied to the model filled with water up to the level of pyloric opening and caused DGR. At the same time, the flow inside the model was recorded with a high speed camera (SVSi, Southern Vision Systems, Alabama, USA) at a rate of 30 frames/sec. After a given time interval, the supply valve was closed and the exhaust valve was opened simultaneously to drain the refluxate.

### Determination of distal pressure causing DGR

To represent variable pressure gradients causing DGR across the pylorus, three different distal pressures of 20, 30 and 40 mmHg (the width of a pressure pulse = 1 second) were determined arbitrarily.

### Measurement of DGR

**Figure 3:**
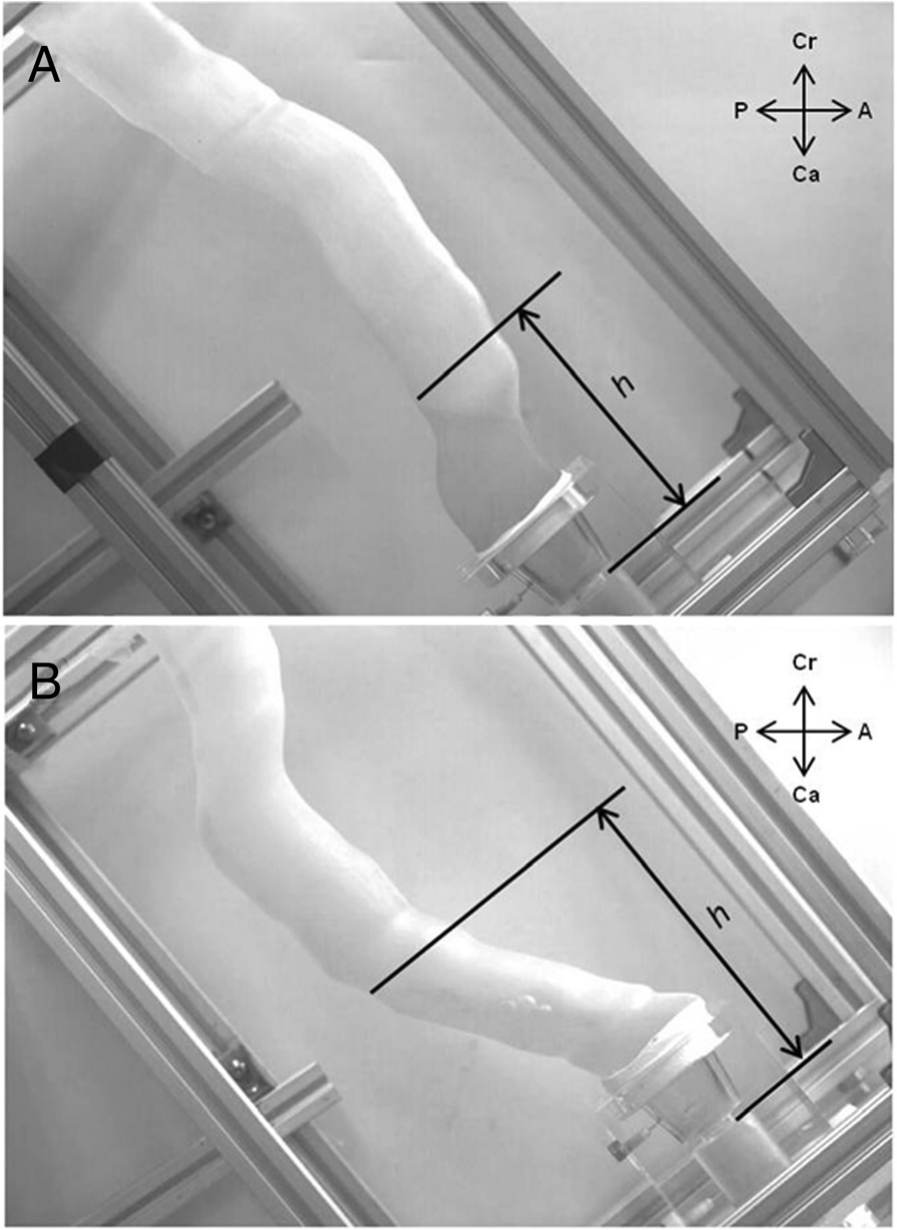
**Gastric tube model in the semi-Fowler position. A**, Retrosternal route. **B**, Posterior mediastinal route. *h,* Maximum height of refluxate; *A*, Anterior; *Ca*, Caudal; *Cr,* Cranial; *P,* Posterior.

After recording the internal flow, the fluid was drained through the exhaust valve to measure the volume. To evaluate the accessibility of the refluxate to the esophagus, the maximum height of the refluaxate (perpendicular distance from the plane of the pyloric opening) was measured by analyzing video clips using a computer-assisted video frame analysis system (MemView 2.1.9, Southern Vision Systems, Alabama, USA) (Figure 3). Accordingly, DGR was measured in 24 cases of 2 routes, 2 pylori, 3 pressure settings and 2 postures. For each case, 10 experiments were conducted to check the reproducibility.

### Statistics

All the data is presented as the mean ± standard deviation. The Mann–Whitney test was used to compare the volume and height of the refluxate between two groups. All analyses were performed using SPSS V12.0 for Windows (SPSS, Chicago, IL) and yielded two-sided p values. Values of p < 0.05 were considered significant.

## Results

The mean volumes and heights of refluxate when the distal pressures of 20, 30, and 40 mmHg were applied for one second to the model in the upright and semi-Fowler positions are shown in Tables 1 and 2.

**Table 1:** Mean volume and height of refluxate according to the size of the pylorus

**Route**	**Posture**	**Pressure (mmHg)**	**Volume (mL)**			**Height (mm)**		
			**Small pylorus**	**Large pylorus**	***p*value**	**Small pylorus**	**Large pylorus**	***p*value**
RS	Upright	20	50.8 ± 4.0	87.8 ± 1.1	.001	72.6 ± 3.6	101.6 ± 4.8	.001
		30	58.4 ± 1.5	150.8 ± 1.1	.008	87.3 ± 3.6	151.4 ± 2.2	.008
		40	69.8 ± 2.6	156.8 ± 3.3	.008	111.0 ± 5.6	166.1 ± 1.7	.008
	Semi-Fowler	20	53.4 ± 4.0	114.7 ± 3.1	<.001	86.9 ± 11.6	137.5 ± 3.9	<.001
		30	58.0 ± 0.7	158.8 ± 0.8	.008	132.7 ± 3.2	181.6 ± 1.0	.008
		40	72.6 ± 0.9	200.2 ± 2.7	.008	135.8 ± 5.5	221.0 ± 3.4	.008
PM	Upright	20	56.4 ± 1.7	96.4 ± 1.7	.001	72.5 ± 5.6	113.4 ± 2.9	.001
		30	61.2 ± 2.2	158.0 ± 3.2	.008	98.3 ± 3.84	165.4 ± 1.5	.008
		40	75.6 ± 1.5	198.0 ± 4.7	.008	118.6 ± 5.0	193.7 ± 6.6	.008
	Semi-Fowler	20	59.0 ± 2.0	144.5 ± 2.8	<.001	164.0 ± 8.0	162.5 ± 5.0	.529
		30	60.6 ± 0.9	176.0 ± 1.8	.004	191.3 ± 9.3	198.8 ± 6.4	.310
		40	73.4 ± 1.3	227.7 ± 4.3	.004	217.8 ± 7.4	233.4 ± 4.2	.008

The width of a pressure pulse was 1 second.

*PM* posterior mediastinum, *RS* retrosternal space.

**Table 2:** Mean volume and height of refluxate according to the route of the gastric tube

**Pylorus**	**Posture**	**Pressure (mmHg)**	**Volume (mL)**			**Height (mm)**		
			**RS**	**PM**	**p value**	**RS**	**PM**	**p value**
Small	Upright	20	50.8 ± 4.0	56.4 ± 1.7	.002	72.6 ± 3.6	72.5 ± 5.6	.684
		30	58.4 ± 1.5	61.2 ± 2.2	.034	87.3 ± 3.6	98.3 ± 3.8	.016
		40	69.8 ± 2.6	75.6 ± 1.5	.008	111.0 ± 5.6	118.6 ± 5.0	.008
	Semi-Fowler	20	53.4 ± 4.0	59.0 ± 2.0	.004	86.9 ± 11.6	164.0 ± 8.0	<.001
		30	58.0 ± 0.7	60.6 ± 0.9	.040	132.7 ± 3.2	191.5 ± 9.3	.008
		40	72.6 ± 0.9	73.4 ± 1.3	.421	135.8 ± 5.5	217.8 ± 7.4	.008
Large	Upright	20	87.7 ± 1.1	96.4 ± 1.7	.008	101.6 ± 4.8	113.4 ± 2.9	.008
		30	150.8 ± 1.1	158.0 ± 3.2	.008	151.4 ± 2.2	165.4 ± 1.5	.008
		40	156.8 ± 3.3	198.0 ± 4.7	.008	166.1 ± 1.7	193.7 ± 6.6	.008
	Semi-Fowler	20	114.7 ± 3.1	144.5 ± 2.8	<.001	137.5 ± 3.9	162.5 ± 5.0	<.001
		30	158.8 ± 0.8	175.6 ± 1.7	.004	181.6 ± 1.0	198.8 ± 6.4	.008
		40	200.2 ± 2.7	227.6 ± 4.8	.004	221.0 ± 3.4	233.4 ± 4.2	.008

The width of a pressure pulse was 1 second.

*PM* posterior mediastinum, *RS* retrosternal space.

### DGR according to the size of the pylorus (pyloric drainage procedure)

For any given route, posture or pressure setting, the mean volumes of refluxate in the large pylorus model were always significantly larger than those of the small one (p < 0.05 for all). The data reflecting the mean heights showed similar tendencies as those of the mean volumes, with the exception of two cases (Table 1).

### DGR according to the route of the gastric tube

For the large pylorus setting, the mean volumes and heights of refluxate in the PM model were always significantly larger than those of the RS model (p < 0.05 for all). The data from using the small pylorus setting showed similar tendencies as those for the large one, with exception of two cases (Table 2).

## Discussion

The controversies about the omission of a pyloric drainage procedure and the use of an extra-anatomical route for minimizing DGR are caused by the limitations of the methodology used in most clinical studies. Most clinical studies have evaluated DGR using various indirect techniques, such as quantification of isotopic markers (Tc99-HIDA) in the gastric or esophageal aspirate [14], 24-hour gastric pH monitoring with the existence of alkaline reflux [15], or using a questionnaire for the reflux episodes [7], etc. All have some potential limitations for clearly verifying the influence of the route of reconstruction or of a pyloric drainage procedure. Apart from the limitations of each method itself, many other conditions that affect DGR, such as the variable intra-abdominal pressure, intestinal motility, posture and the physical activity of the patients during the study period may interfere with the results of studies.

It is for this reason that we designed the present study. Our goals were to evaluate the amount of refluxate and the esophagus’ exposure to refluxate purely according to the surgical factors. Additionally, we thought excluding or controlling the variables which are highly variable in a real clinical situation was essential in the design of a testable model. Therefore, we adopted the ‘flow visualization model’; a gastric tube where the internal flow can be easily accessed and directly observed. To simplify the comparison for this study’s purpose, we made the tubes cylindrical with circular cross sections of the same diameter. The diameter of the pyloric opening after drainage procedure was determined arbitrarily, doubling that of the original pyloric opening [13]. Because any reference values of pressure gradient causing DGR across the denervated pyloric sphincter could not be found in the literature, they were determined according to duodenal manometric data in an upper gastrointestinal motility study of healthy volunteers [16]. In that study, the amplitude of contractions in the duodenum was in the range of about 22 to 27 mmHg during fasting and after the meal. In addition, because it has been established that especially after abdominal surgery intra-abdominal pressure can be elevated (5 ~ 15 cmH_2_O) promoting DGR [17], the pressure gradient settings were set as 20, 30, and 40 mmHg in this study. The width of a pressure pulse was also determined arbitrarily because there were no reference values available in the literature.

In the literature, a pyloric drainage procedure is known to have an unclear effect on reflux [3-5,9,10]. In the present study, the degree of DGR had the definitive tendency to be higher in a gastric tube with a larger pyloric opening. In terms of fluid mechanics, DGR can be expressed as a process in which the developed pressure gradient across the pyloric opening becomes zero through the pressure energy loss (which may be transformed into other types of energy). In our model, the pressure energy loss occurs through two-step process (in the pyloric opening, and then in the gastric tube itself). In the 1st step, the pyloric opening can be considered an orifice existing in a pipe. The analytical solution of the pressure energy loss Δ*p* across the pylorus can be given by solving the Bernoulli obstruction equation as follows [18]:

$$\mathrm{\Delta p}=\rho Q^{2}\left(1-\beta^{4}\right)/2C_{d}^{2}A_{t}^{2}$$

where *ρ* is the fluid density, *Q* is the flow rate through the pipe of diameter *D*, *β* is the opening ratio of the orifice *d* (=*d*/*D)*, *C*_*d*_ is the discharge coefficient, and *A*_*t*_ is the opening area. If the opening ratio *β* becomes smaller (that is, pyloric opening gets smaller), the pressure energy loss is increased because (1-*β*^4^) gets larger and *A*_*t*_ gets smaller. In addition it is well known that *C*_*d*_ also get smaller for the smaller *β*[18]. This means that in small pylorus setting, more pressure energy is exhausted in order to pass the small opening, and less energy is transformed to cause reflux, compared to the large pylorus model. This can be an adequate explanation for the results of the pyloric drainage procedure in the present study.

Another important surgical issue for DGR is the route of esophageal reconstruction. In the present study, the degree of DGR had the definitive tendency to be higher in a PM gastric tube. In terms of fluid mechanics, this issue is related to the 2nd step of reflux (in the gastric tube itself). In the 2nd step, the pressure energy loss mainly occurs by gravity and wall friction in the gastric tube as follows [18]:

$$\mathrm{\Delta p}=\mathrm{\rho gh}+f\frac{L}{D}\frac{\rho v^{2}}{2}$$

where *g* is the gravitational acceleration, *h* is the vertical height of refluxate from the horizontal line, *f* is the Darcy friction factor, *L* is the length of refluxate from the pylorus, and *v* is the fluid velocity. At the early stage of reflux, the influence of gravity is important and relatively straightforward, and it can be also estimated intuitively. As shown in Figure 1 and Figure 3, the RS tube is more upright than PM tube in the lower portion of gastric tube. This aspect is exaggerated when the tube is tilted into a semi-Fowler position. Therefore, more pressure energy is consumed to overcome the gravity in RS tube than in PM tube, since the former is more influenced by gravity due to its upright shape. This explanation may be used to explain the results of most cases in our study. However, about the influence of wall friction, which becomes significant as refluxate ascends, further intuitive estimation becomes difficult because the friction factor should be directly measured in a diverse environment. Thus, the pressure energy loss in the upper portion of gastric tube can vary greatly according to the shape and its course. In a few cases, we think that the wall friction may have played a role in our inability to show a significant difference.

There are some limitations in this study. The first relates to the design of the model. The gastric tube model was highly simplified intentionally for the purpose of this study. In a more sophisticated model which mimics a true gastric tube, we would have to take into consideration factors such as possible contractility of the gastric tube and remnant esophagus, and environmental factors such as pulsation of the heart or the great vessels, to name only a few. The influence of negative intrapleural pressure is another important factor that may promote DGR and was not demonstrated in our model. These criticisms are somewhat countered by the fact that the intrathoracic stomach likely functions as a passive conduit without any contractility for a considerable period, and all the factors except the curve of the tubes and the size of pylorus were the same among the compared groups. We also do not think the issue of negative intrapleural pressure weakens our conclusion because the effect of negative intrapleural pressure would be more definitive in a PM gastric tube than in a RS tube. The second limitation of our study is that the pressure settings were set arbitrarily. Actually, when DGR occurs in patients, it is difficult to know how high the pressure gradient develops, and how long it persists. However, because this study focused on the difference in the dynamics of the refluxate according to the morphology of the tubes, it is not problematic to apply a certain set of distal pressure with a short duration as long as same conditions are applied to the compared groups. The third limitation is whether the conclusion of the present research study is clinically meaningful enough to make a change in a clinical practice. Although statistically significant, the actual differences in the amount and height of refluxate were small between different routes of reconstruction (Table 2). It is not clear whether these relatively minor differences have significant clinical relevance. When discussing a clinical relevance issue, we should consider gastro-esophageal reflux (GER) as well, which was not demonstrated in our model. As shown in the present study, it is clear that a pyloric drainage procedure promotes DGR and in turn, increases bile reflux into the stomach and the esophagus. On the other hand, as shown in our previous work [13], it facilitates gastric emptying and therefore may reduce GER. Thus, it is hard to confirm whether the omission of a pyloric drainage procedure could reduce the patients’ reflux symptoms. Future studies of reflux in the clinical setting, in correlation with symptoms, endoscopic and histological findings, may be required to get more comprehensive information.

## Conclusions

We established a method to observe and measure DGR inside the intrathoracic stomach as an esophageal substitute by devising the flow visualization model. Using this model, we have demonstrated that the degree of DGR was significantly higher in the intrathoracic stomach located in PM and/or with a pyloric drainage procedure being performed in the fluid mechanics. This finding may help reduce the incidence of DGR after esophagectomy with gastric interposition in real-world clinical settings.

## Abbreviations

DGR: Duodenogastric reflux; RS: Retrosternal space; PM: Posterior mediastinum; GER: Gastro-esophageal reflux.

## Competing interests

The authors declare that they have no competing interests.

## Authors’ contributions

CP: literature research, data acquisition, data analysis, manuscript preparation, manuscript editing; JL: study concepts, study design, definition of intellectual content, literature research, data analysis, manuscript preparation, manuscript editing; JS: study design, development of experimental model, data acquisition, manuscript editing; SC: design of experimental setup, experiment, data acquisition; KK: data analysis, statistical analysis, manuscript editing. All authors read and approved the final manuscript.
